# Industrial IoT, Cyber Threats, and Standards Landscape: Evaluation and Roadmap

**DOI:** 10.3390/s21113901

**Published:** 2021-06-05

**Authors:** Lubna Luxmi Dhirani, Eddie Armstrong, Thomas Newe

**Affiliations:** 1 Confirm Smart Manufacturing Research Centre, V94 C928 Limerick, Ireland; thomas.newe@ul.ie; 2 Department of Electronic and Computer Engineering, University of Limerick, V94 T9PX Limerick, Ireland; 3 Johnson & Johnson, Advanced Technology Centre, University of Limerick, V94 YHY9 Limerick, Ireland; earmstr1@its.jnj.com

**Keywords:** industrial IoT, cybersecurity, threats, standards, IT/OT, industry 4.0, IoT/M2M

## Abstract

Industrial IoT (IIoT) is a novel concept of a fully connected, transparent, automated, and intelligent factory setup improving manufacturing processes and efficiency. To achieve this, existing hierarchical models must transition to a fully connected vertical model. Since IIoT is a novel approach, the environment is susceptible to cyber threat vectors, standardization, and interoperability issues, bridging the gaps at the IT/OT ICS (industrial control systems) level. IIoT M2M communication relies on new communication models (5G, TSN ethernet, self-driving networks, etc.) and technologies which require challenging approaches to achieve the desired levels of data security. Currently there are no methods to assess the vulnerabilities/risk impact which may be exploited by malicious actors through system gaps left due to improper implementation of security standards. The authors are currently working on an Industry 4.0 cybersecurity project and the insights provided in this paper are derived from the project. This research enables an understanding of converged/hybrid cybersecurity standards, reviews the best practices, and provides a roadmap for identifying, aligning, mapping, converging, and implementing the right cybersecurity standards and strategies for securing M2M communications in the IIoT.

## 1. Industrial IoT (IIoT)

Industrial IoT (IIoT)/Industry 4.0 (I4.0) [1] is a concept of an innovative and intelligent fully connected factory implementing disruptive technologies (i.e., IoT, cloud computing, artificial intelligence, etc.) and innovative solutions (IIoT, automation, monitoring, etc.) collectively enhancing the production environment with lower costs, agility, efficiency, remote operations, etc. In such an autonomous environment, data and network security are key driving factors. With the growing implementations of IIoT applications and services, the spectrum of cybersecurity threats has changed with it and requires enhanced security measures and controls to be developed [2,3,4]. The threats and types of breaches possible on the internet during the pandemic must not be underestimated. Breaches happening in the industrial IoT domain would be critical due to specific exposures that are related to machine-to-machine (M2M) communication and environments [3]. M2M communication networks are an integral part of connected factories involving high dependency on next generation wireless communication systems (5G, time sensitive networking, etc.) [4,5] and involving self-automated, self-driven, and self-learning network characteristics. The future M2M devices are anticipated to work independently and make decisions based on artificial intelligence and machine learning algorithms. These types of devices where human intervention will be minimal require high levels of operational security because they may cause disruptive hazards [3]. The main source of threats for M2M communications comes from unanticipated breaches arising from the internet, software which are mostly identified post implementation, limited capabilities due to low-energy, cost, remote locations, bandwidth, legacy systems, etc. There is a substantial gap between the existing information technology and operational technology (IT/OT) domains which makes the IIoT environment more vulnerable to existing security issues [5,6,7,8,9,10]. With billions of IoT/M2M devices connected in the industrial environment, it may potentially create multiple weak-entry points and lead to compromised assets/information/privacy issues. Without appropriate standards and security controls in place, it will be hard to classify the cyber threat impact and the information altered/manipulated. Identifying the breach before damage has incurred is critical to the whole environment [5].

With the advanced use-case implementations (i.e., smart industrial robotics, digital twins), data analysis and cybersecurity are essential for securing the vertical industries. Bridging the gap between the security metrics will facilitate end-to-end (E2E) security, attack tolerance and early detection of attacks [9] mitigating potential incidents on the I4.0/industrial control systems (ICS). The novelty of this research lies in the following: (i) advances knowledge in this field by deep diving into cybersecurity standards, (ii) provides insights for designing/converging IT/OT security architectures, (iii) emphasizes the necessity of implementing interoperable and hybrid standards connecting different complex interfaces, (iv) bridges the IT/OT divide, and (v) provides a strategic alignment mitigating IT/OT cyber risks in the IIoT/I4.0.

**Figure 1:**
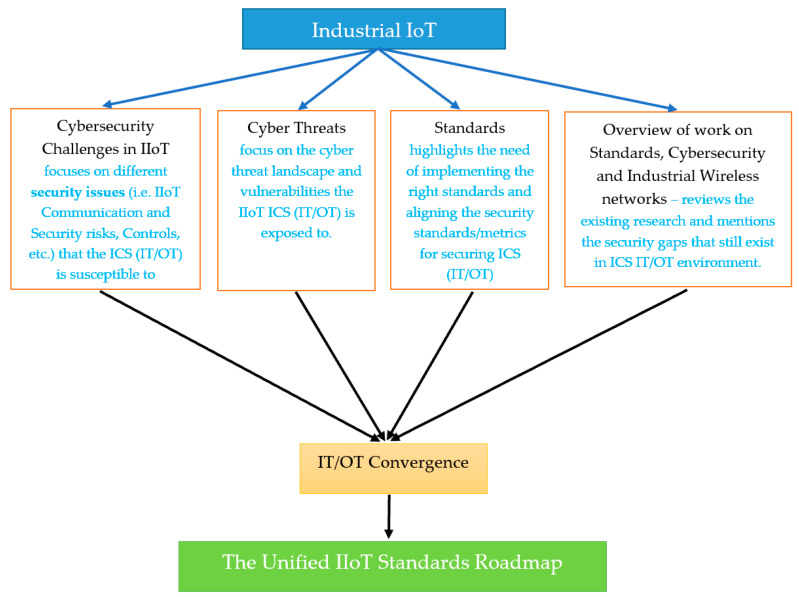
Rationale.

Figure 1 presents the scope, structure, and objectives of this research.

This paper is structured as follows, Section 2 outlines cybersecurity challenges in IIoT, Section 3 discusses cyber threats in the context of industrial control systems (ICS), Section 4 articulates the scope of different security and communication standards and how alignment can be achieved. Section 5 presents the author’s analysis on previous published journal, conferences, and white papers relevant to the scope of this research. Section 6 investigates IT/OT convergence issues in detail, followed by Section 7 which provides a roadmap to a unified standard framework mitigating the cyber threats and standardization issues that arise in the I4.0/IIoT environment due to IT/OT convergence gaps. 

## 2. Cybersecurity Challenges in IIoT

This section focuses on different security issues (i.e., denial of service, data theft, manipulation, eavesdropping, etc.) [8,9,10,11]. The authors relate these concerns to the IIoT/I4.0 domain and highlight how these unresolved issues [12,13] leave the industrial environment susceptible to security breaches.

### 2.1. Security Issues

#### 2.1.1. IT/OT (Data Security Issues)

Cyberattacks at the operational technology (OT) level have grown considerably recently, as it involves integrating new interfaces (i.e., IT systems, cloud, etc.) [14,15] providing flexibility and remote access to both new/old OT (i.e., SCADA, PLCs, etc.) systems. One of the reasons for the increased awareness in this domain is a significant increase in ICS cyberattacks (i.e., the USA’s largest fuel-pipeline ransomware attack) [16]. Additionally, proprietary production knowledge becomes an IT security problem [3] with IIoT exposed to various types of cyber threats due to its dependency on new communication models and devices. The OT domain’s major focus depends on availability and integrity of ICS whereas the IT sector focuses on confidentiality and integrity of applications, services, and supporting technologies. Lack of convergence between IT and OT systems develops knowledge gaps [17] allowing sophisticated and targeted cyberattacks to take place. 

#### 2.1.2. IIoT Communication and Security Risks

IIoT and machine-to-machine (M2M) communications characteristics depend primarily on high throughput and low latency, and utilize new communication technologies such as 5G, Wi-Fi 6, and Time Sensitive Networking (TSN) [4]. These new communication models and devices expose the IIoT environment to a wide range of cyber threats. With the increased usage of IoT-based devices, assessing, monitoring, and securing the endpoints of these devices is subject to various security-based challenges (e.g., lack of standard regulations for all IoT devices from different vendors [9,10], trusting third-party cloud vendors). Besides this, there are a number of other risks associated with securing the M2M communication environment such as [10,18,19,20] poorly configured devices, malicious IoT node injection, and standardization of M2M communications (IoT, cloud, edge, etc.). The sensitivity of compromised M2M devices may be assessed on the basis of the impact type, i.e., materialization of a threat leads to compromised confidentiality, and/or availability, and/or integrity of the network, and the impact factor/scale, in terms of users, downtime duration, number of cells affected, sensitivity of the information altered/accessed, etc.

#### 2.1.3. Security Controls and Standardization

Security standards and security controls enable the industrial environment to implement the best security practices available and make it easier to identify a breach when it takes place as the risk factors associated [2] are known and thereby can reduce the incident response and recovery times. Other benefits of applied standards are discussed below.

Visibility, insights, and control are critical for the fully automated vertical model which relies on ultra-reliable low latency communications (URLLC), massive machine type communications (mMTC), and enhanced mobile broadband (eMBB) [2]. With such M2M service requirements, standards and security controls can grant transparency in assessing the potential security risks at the production levels.

The majority of industries put commercial standards (security controls, IT/OT standards, communication standards, etc.) [21] into practice without understanding and evaluating their production environment. This can develop disparities and leave the IT/OT domains vulnerable to cyber threats and data security vulnerabilities. With escalating cyberattacks (i.e., malware, phishing, etc.) on the ICS environment, it is necessary to lay down a defensive approach for identifying the threat actors. Threat intelligence frameworks provide visibility and classify threats based on predefined metrics [22,23]. They play an integral role in securing I4.0, however, complete reliance on a certain framework or model is not always advisable to provide a comprehensive security strategy. Outdated and legacy systems deployed in the production environment reduce the efficiency and impact of security controls. Legacy systems with custom configurations, root of trust, identity protocols, etc. add complexity to setting up new security controls to old interfaces and securely extracting data.

A fully connected factory involves different applications, services, communication models, and cloud structures. Service level agreements [1] are of high importance for assessing, aligning, and controlling the security, privacy, and quality of service (QoS) metrics associated with the I4.0 environment. Situations where SLAs are not assessed and monitored properly may lead to unexpected service disruption, downtime, and third-party subcontracting vendor issues [4].

Around 48% of manufacturing industries have suffered a cyberattack, and as per InfoSys [24] 89% of manufacturing sectors understand the importance of data standards but only 11% of them invest in implementing relevant security controls and standards. Failure to meet the desired security levels and standards makes it hard to classify the threat impact and potential breaches.

**Figure 2:**
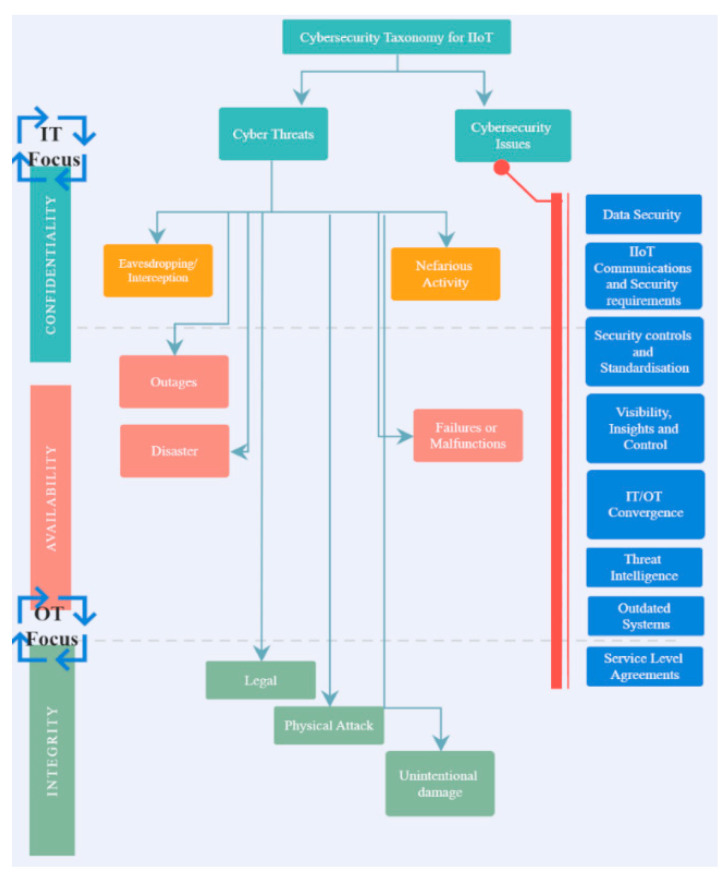
Cybersecurity taxonomy for IIoT.

The IIoT will streamline manufacturing sectors where the focus will shift to integration, digitization, and management of all physical resources through a uniform and flexible approach, making the cybersecurity landscape even more challenging, since the potential damages incurred on the production environment due to a breach could be serious. The security attributes (confidentiality, integrity, and availability (CIA)) provide ways to assess and benchmark data and network security [3]. The CIA taxonomy is important because it meets the needs of wireless M2M networks regarding their specific security issues [25] (as shown in Figure 2).

Cybersecurity risks within the European member states are analyzed based on the ISO/IEC 27005:2018 [2,26] risk assessment methodology which takes the following parameters into consideration: the types of threats posed to wireless networks, threat actors, core assets and their level of sensitivity, vulnerabilities, risks, and related situations. With the augmented use of IoT-based devices (10–100 x more) and technology, the number of cybersecurity threats to supply-chain management will increase as well. Besides the data security CIA triad, within the IoT-based environment there is another attribute that plays a vital role, freshness of data [3]. This feature (if deployed) will provide insights on the time duration of data-sent/received and assist in assuring if it has been compromised or tampered within the defined timespan.

## 3. Cyber Threats

**Figure 3:**
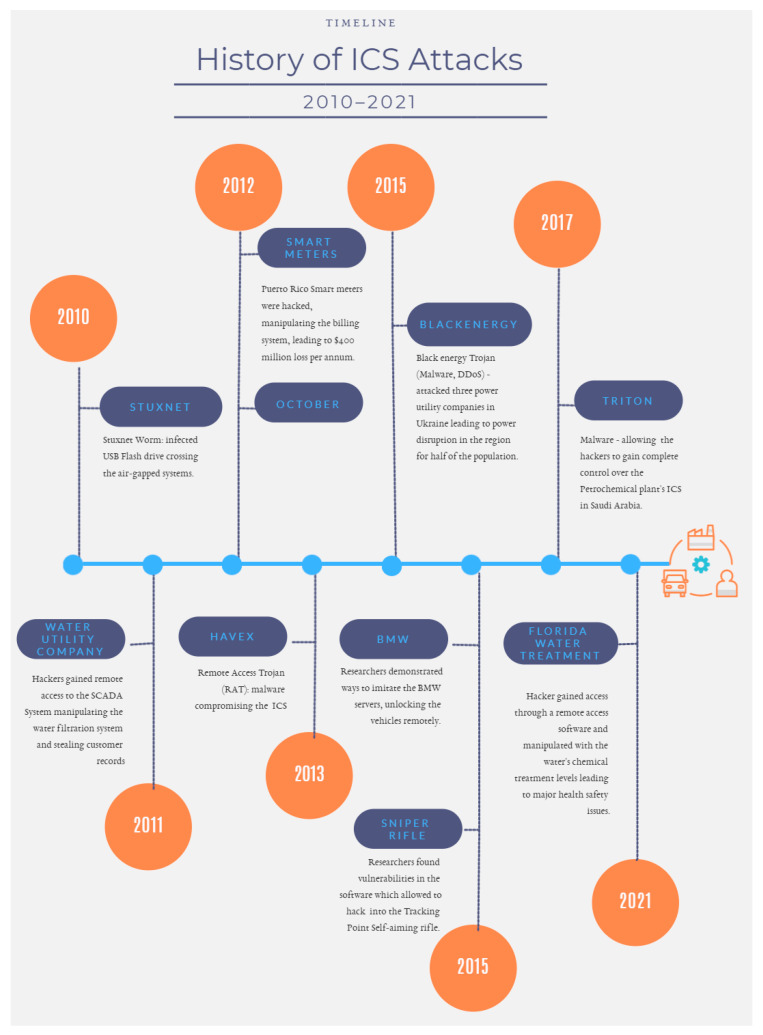
Timeline and history of ICS cybersecurity attacks.

Cyber threats involve malicious acts of stealing, manipulating, or disrupting data (exploiting data confidentiality, integrity, and availability). These malicious acts are executed using various methods [3]: viruses, malware, denial of service (DoS), eavesdropping, etc. and may be carried out by people with distinct abilities and classes (i.e., hacktivists, state-sponsored, knowledgeable insider, organized crime, hackers, amateur, etc.). Cyber threats may expose industrial control systems (ICS) actively or passively. Active threats are easy to identify compared to passive threats [19] where hackers use backdoors for eavesdropping which is hard to identify and can affect the reliability and integrity of a system. Figure 3 presents attacks [19] where industries endured substantial losses as a result of lack of control, modified information, malware, keyloggers, etc., active and passive cyberattacks.

In recent times, there has been a spike in the IT/OT cyber attack surface (malware, spyware, phishing attack, ransomware, zero-day attacks, etc.), and with the implementation of new communication models and standards the impact of these threats and risk needs to be assessed.

As previously mentioned IIoT/I4.0’s M2M communication relies on distinct requirements, such as URLLC, mMTC, and eMBB [2] which enable real-time data with low latency and high reachability. These networks (5G, Wi-Fi 6, etc.) are fully equipped to support the key requirements but what is important to note is that any IoT/M2M gaps identified and exploited by hackers at this point can lead to high-risk threats (i.e., outages, failures, nefarious abuse, etc.) as shown in Figure 2 and Figure 3. To mitigate such risks, it is recommended to thoroughly understand and implement cybersecurity standards [27,28]. However, only a handful number of industries implement them effectively and fewer again understand their existing security maturity level when aligned with the industry’s current risk level. These steps are essential to improve ICS’s ability to identify, predict, and prevent cyber threats. 

The European Union Agency for Cybersecurity (ENISA) [2] provides a threat landscape classifying the threats/vulnerabilities, network assets, and sensitivity, which the IIoT may be exposed to using 5G networks. This model also leverages from and complements information by providing a more detailed technical view on the 5G architecture, sensitive assets, cyberthreats affecting the assets and threat agents [29]. The information produced for the threat landscape is based on publicly available information published by 5G standardization groups and bodies (i.e., ETSI, 3GPP) [30,31] and 5G investors such as operators, vendors, and national and international organizations.

Though ENISA’s threat landscape assists in anticipating the possible threat scenarios (as shown in Table 1), it is important to note that with limited 5G IIoT/I4.0 use-case implementations there would still be various threat vectors and inconsistencies which are yet to be identified. These gaps cannot be fully eliminated merely by implementing the existing cybersecurity standards due to various factors such as lack of alignment, mapping, and understanding the production environment itself. The actual types and number of cyber threats may only be visible as the use-case deployments in I4.0 progress.

The majority of the threats classified to fall under the high-risk category meaning that any disruption or vulnerability exploited at this stage may lead to compromised security services. Threat classification and threat intelligence models assist the manufacturing sectors in being proactive and prepared for worst-case scenarios and build backup solutions and contingency plans for disaster situations. In order to regulate the security services, standards, security controls, and frameworks assist in devising a security strategy and mitigation plan which is discussed in the next section.

## 4. Standards

**Figure 4:**
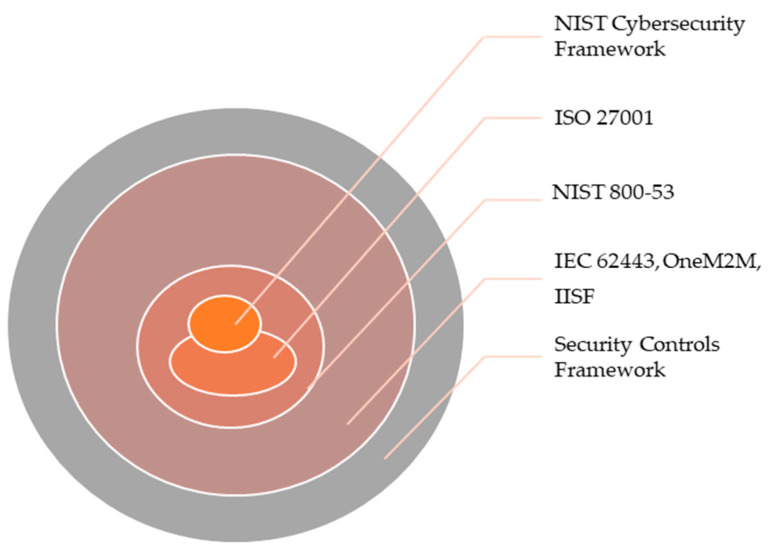
Hybrid security standards and controls adapted from [40].

Cybersecurity standards are the best practices for information security, secured communications and are generally applicable to all sectors. Considering the IIoT/I4.0 environment, a wide range of standards are implemented such as IEC 62443 (cybersecurity), ISO 27001 (information security), NIST 800-53 (Rev 4 and 5)—control baselines, NIST 800-82 (Rev 2)—securing ICS, industrial internet security framework (IISF) [32,33,34,35], ENISA, etc. As discussed earlier, the standards (3GPP, IEEE 802.11ax, and 802.1) [36,37,38] supporting the novel communication technologies need to be aligned and evaluated using as common threat landscape. IIoT/I4.0 falls into horizontal and vertical categories based on their service sector (i.e., healthcare, transportation, energy, etc.) [39]. These sectors implement different IoT/M2M communication standards. However, the authors have specifically focused on the IIoT/smart manufacturing industry for this research. The versatility of I4.0 has been discussed before and such models require various combinations of security standards (see Figure 4 adapted from [40]).

Implementing hybrid standards is a common practice in the manufacturing environment but what the majority of industries fail to understand is the role of the standard. Each standard may have something unique, but may also have its own set of limitations. As an example, if hybrid standards are compliant and fill the gaps that may be an advantage but if they overlap developing complexity that may be an issue, as it will be difficult to analyze and assess the security metrices.

The conformance disparities addressed in [39] are classified in the following categories: technical differences (i.e., communication range, QoS, protocol layers, etc.), policies (i.e., international and regional), and business drivers. This means that standards need to be adaptable and cover security from both technical (incident response, risk management, vulnerability management, cybersecurity policies, etc.) and non-technical aspects.

### 4.1. Standards Complementing or Comparative

Each security standard may apply to similar or different aspects of information security and controls (see Table 2 below).

**Figure 5:**
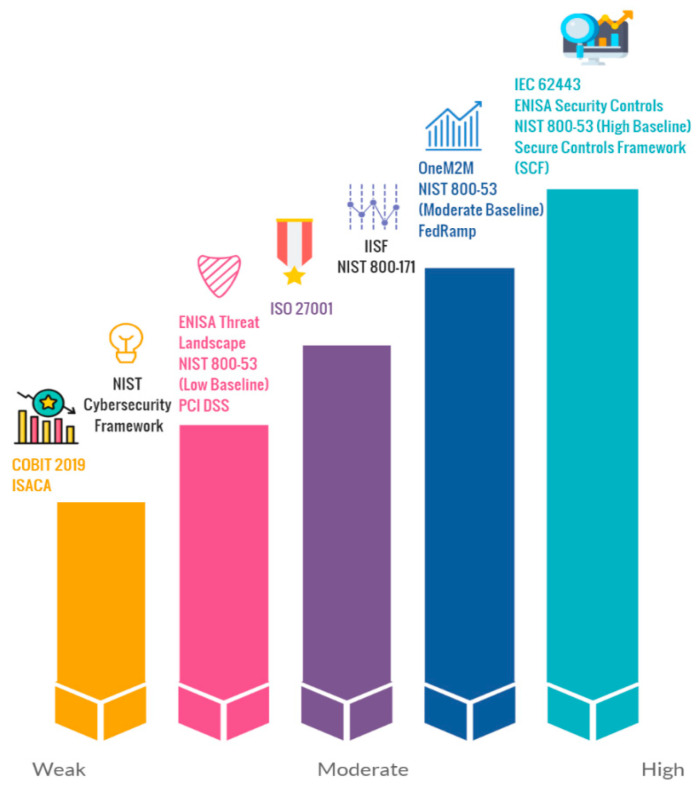
Cybersecurity standards coverage adapted from [40].

Above are some examples for comparing different data security standards. These can be further classified at the performance (coverage), effectiveness, and metrics levels as well. The authors designed Figure 5 (remodeled from [40] based on the scope of this research) to present different cybersecurity standards classified by impact and security coverage. It is important to note that even if some industries have the same operations, they may differ in terms of their security strategy and controls. This is necessary because risk classification and security metrics need to be aligned with the industry’s business needs. Based on past attacks on IIoT ICS (shown in Figure 3) where Black Energy and Triton [46] affected huge amounts of manufacturing industries, it has become necessary to implement different security strategies and services to protect the IIoT environment. 

An interesting point at this stage would be linking the standards with the threats addressed in Section 3. ICS metrics are bound by high-level objectives. These objectives are closely monitored and controlled. If they fail to meet the desired level of security, there may be underlying issues that need to be addressed. An objective may involve restricting physical access to the ICS networks and devices [43]. The aim of this objective can be assessed by monitoring any unauthorized access granted or occurred. Unauthorized access has been discussed in ENISA’s threat classification (see Table 1). Another security objective may be to restrict logical access to the ICS and network activity. This type of security measure falls under encryption techniques and the identity and access management (IAM) category (mentioned in Table 1), protecting the ICS components from being exploited by malicious actors. This type of objective can be mitigated by analyzing the nefarious abuse/activity (stated in Table 1). Each of the threats and vulnerabilities discussed may lead/link directly or indirectly with the cybersecurity controls and standards [47]. All industries may have different objectives set for measuring their security controls which need to be mapped with the standards. Many enterprises fail to map these metrics and implement tools like Zivver’s [48] to assist them. However, tools may only assist in mapping the existing objectives but cannot distinguish the security disparities.

### 4.2. Standardization Bodies

Standards play a vital role in enhancing the visibility, conformance, monitoring and control of an industrial environment. Since I4.0 is expected to be a fully connected and autonomous industry, it may implement standards supporting different processes such as IT/OT, M2M communications, telecommunications, and networking standards, etc. These standardization bodies (3GPP, IEEE, IEC, etc.) [30] have been working together to standardize the future communication networks (i.e., 5G, Wi-Fi-6, TSN ethernet) [36,49] with the aim of supporting M2M applications/devices/communications in the IIoT/I4.0 environment. These standard solutions must fully comply with M2M/IoT performance and security requirements creating a safe functioning and credible environment. The well-known standards related to IIoT M2M communication networks/devices are outlined below. 

#### 4.2.1. 3rd Generation Partnership Project (3GPP) 

5G’s network architecture implements the 3rd Generation Partnership Project (3GPP) standard and is equipped with radio access, core transport networks, and service capabilities, providing complete system specifications required to support IIoT [50,51]. 5G services are provided through a generic framework to network functions (i.e., modularity, reusability, self-containment, etc.) which are authorized to use these services. The 5G core architecture also defines 5G’s support specifications to utilize the cloud and service-based architectures overlapping technical functionality (i.e., security, etc.) [52]. It also assists the architectural principles of integrating network function virtualization (NFV) functions using the multi-access edge computing (MEC) infrastructure [50,53].

Based on the 3GPP standard in comparison to former networks, the URLLC range has been reduced to 1 ms and provides additional features such as end-to-end security (99.999%). The 3GPP Release 15 considers three basic types of service (i.e., eMBB, mMTC, and URLLC) and by utilizing slicing these services could be separated in an ideal way with respect to performance metrics such as latency, throughput, and availability/resilience, and allow each slice to cover different use-case requirements. The 3GPP standard [30] combines validation for splitting verification from access points, using extensible authentication protocols (EAP) to provide secure communications, introducing flexible security policies to address more use cases, and subscriber permanent identifiers (SUPI) to ensure network privacy concerns [54]. With the increasing number of virtualized nodes, the complexity arises while evaluating and assessing the E2E (end-to-end) security of 5G applications, devices, and architecture. The most common IoT-based risks addressed to date [55] have been associated with insecure web, mobile and cloud interfaces, lack of suitable authentication/validation/encryption system, and privacy and security concerns.

In a fully automated industrial environment, security is a key aspect and needs to be managed proactively to offer reliable data security, integrity, and authenticated services. While 5G follows the 3GPP norm which defines the core and access functions and assessment criteria, it fails to fully incorporate all security aspects (i.e., transport and transmission functions, and internetwork exchanges) leaving the environment vulnerable to security-based risks. Looking at the future, it is likely that the growth in IoT communications will actively move toward 6G [56,57] which provides disruptive communication technologies and innovative network architectures. Telecom providers currently working on standardizing the 5G network will soon be following with 6G as the need for fast, reliable, and autonomous communications grows.

#### 4.2.2. ETSI OneM2M

OneM2M, developed by the European Telecommunication Standards Institute (ETSI) [31], provides a unified standardized end-to-end M2M architecture for vertical industries. The oneM2M standardization partnership includes various M2M standardization bodies such as ETSI M2M, CCSA, TIA TR-50, TTA, ATIS, ARIB, and TTC [31] for the M2M/IoT environment. The core idea for this framework is to enable uniform secure data sharing, applications, interoperability, scalability, and connecting massive M2M devices to support IIoT/I4.0. OneM2M’s network domain supports the existing standards and technologies such as 3GPP, IETF, and TISPAN. The M2M gateway and device domain M2M area networks are based on DLMS, CEN CENELEDC, PLT, and ZigBee [31]. ETSI’s M2M has transitioned from independent silos to a standardized approach [51]. ETSI’s M2M network architecture is implemented worldwide and involves the following principal components [30] M2M device domain, M2M communications domain, and M2M service/application domain. ETSI has put in noticeable efforts in developing E2E architecture, protocols, and interfaces for supporting M2M communications. ETSI’s guidelines for IIoT cybersecurity acts as a roadmap for improving the threat landscape in the industrial/cyber–physical systems environment [51].

#### 4.2.3. ISO/IEC 27033:1:2015

This standard provides guidelines for IS/IT network security and management and is widely implemented in the IIoT/I4.0 environment. It defines measures to identify, assess and benchmark a network and its security metrics [56]. QoS, which is an essential part of network security, can be accomplished by implementing ISO/IEC 27033:1:2015. “*It also introduces how to achieve good quality technical security architectures, and the risk, design and control aspects associated with typical network scenarios and network technology areas*” [33,58]. This standard in alliance with other standards complements the overall industrial IT domain.

#### 4.2.4. IEC 62443

The IEC 62443 is the de facto standard for cybersecurity in industrial control systems (ICS), as it is the only one being applied internationally and cross-industry [46]. It is defined by the IEC in cooperation with the International Society for Automation (ISA). IEC 62443 provides methods to manage risks related to cybersecurity threats in an automation environment/IIoT paradigm [32]. The IIoT/I4.0 environment involving IACS is subject to the CIA metrics. In [24,42,55] the authors state that the existing standards were not aligned/designed with the challenges associated with M2M cybersecurity. One of the biggest concerns with this standard is to align (making it compliant) in cross zone communication areas and software updates. With the emergence of IIoT, this architecture is no longer the norm, and the development has accelerated an already ongoing convergence between IT/OT that results in an increase of the IACS attack surface [59]. There is an apparent risk that the introduction of IIoT makes parts of the standards outdated [32].

#### 4.2.5. IEEE

The IEEE working group (WG) [36,37,38,60,61] are actively working on improvements and standardization of communication technologies supporting IIoT use cases. The 802.11 WG has introduced new/revised standards to support the IIoT requirements and 802.11 and cellular radio technologies are largely complementary in meeting the comprehensive 5G service vision. WLAN access is an integral part of the 5G system architecture developed by 3GPP. This provides the flexibility so that both core network anchoring and the RAN based anchoring from 4G systems are seamlessly supported in 5G system architecture.

#### 4.2.6. NIST

The National Institute of Standards and Technology (NIST) standards [43,62] are based on the best course of action and security controls to assist manufacturing industries to meet specific regulatory compliance requirements. Different NIST standards are implemented in IIoT, such as NIST 800-53, NIST 800-82, and NIST Cybersecurity Framework [43,44]. Each of these standards differentiates with regards to the security controls and risk management aspects. NIST complements the IIoT when deployed alongside other security standards.

#### 4.2.7. Industrial Internet Security Framework (IISF)

The Industrial Internet Security Framework (IISF) [35,63,64] is a cross-industry-focused security framework comprising expert vision, experience, and security best practices. It was designed to enable IT/OT convergence and set the architectural framework and direction for industrial internet. The IISF focuses on the following characteristics [64]: safety, security, reliability, resilience, and privacy. IISF gives an in-depth sight of risk management and assessment. “*IISF reviews security assessment for organizations, architectures and technologies. It outlines how to evaluate attacks as part of a risk analysis and highlights the many factors that should be considered, ranging from the endpoints and communications to management systems and the supply chains of the elements comprising the system. Different roles are identified that should be considered in conjunction with the key characteristics, including, owner/operator, system integrator/builder and equipment vendor. Each role offers different risk management perspectives that affect the decisions regarding security and privacy*” [64]. From a functional point of view, IISF separates security evaluation into endpoint, communications, monitoring and configuration building blocks with subdivisions for each one. Each perspective offers implementation of best practices. The IISF breaks the industrial space down into three sections: (i) component builders (create hardware and software), (ii) system builders (combine hardware and software solutions), and (iii) operational users (manage the risk to their industrial processes posed by the systems). To ensure E2E security, industrial users must assess the level of trust worthiness of the complete system. “*IISF builds on the IIoT Reference Architecture (IIRA), that lays out the most important architecture components, how they fit together and influence each other*” [63].

#### 4.2.8. Threat Intelligence

ICS and IT systems are widely intermingled, but the IT/OT teams do not fully understand or address each other’s operating concerns, leaving system, control, and process gaps [17,65]. The ICS risk impact may increase with I4.0 due to networked systems, remote access and management, and wireless communication in process control combined with increased usage of mobile devices. Insights, visibility, and control are essential for securing M2M communications in I4.0. Besides implementing different security and communication standards, threat intel assists enterprises in being proactive, assessing and analyzing the risk factor, allocating resources and understanding the threats relevant to I4.0. The MITRE ATT&CK framework [22] and IBM X-Force [40] are well-known threat intelligence tools, widely implemented for designing a security strategy and evaluating the threat landscape.

MITRE developed the ATT&CK framework for documenting and tracking various techniques which attackers may use through different stages of a cyberattack and to infiltrate the industrial network and exfiltrate sensitive data. ATT&CK stands for adversarial tactics, techniques, and common knowledge. The framework is a matrix of different cyberattack techniques sorted by different tactics [22]. ATT&CK also supports governance, risk management, understanding of attacker behavior, methods for classifying and mitigating threats, adversaries, and the potential methods that attackers may use to compromise the industrial network [66]. Other benefits of implementing this framework include: (i) mapping IEC 62443 controls to the MITRE ATTACK ICS Profile, (ii) insights (on initial access (e.g., wireless or supply-chain compromise)), execution (e.g., man-in-the-middle), persistence (e.g., project file infection), evasion (e.g., masquerading), and discovery (e.g., network sniffing, lateral movement, collection, command and control, inhibit response function, impair process control, etc.) [66].

IBM X-Force [23] is a cloud-based threat intel platform that works in collaboration with other technology industry partners, sharing common goals of collecting and sharing threat intelligence. It provides insights on the latest threats, finding suitable actions against threats and seeking expertise from security consultants. Some of the features provided by X-force are incident response time and threat intelligence services, security information and event management (SIEM), and security orchestration, automation and response (SOAR) which assists in using existing tools for optimizing incident response.

The MITRE framework provides a proactive edge, predicting risks and alignment with the IEC 62443 security standard, whereas X-Force offers a defensive tactic but provides sources for actionable intelligence on ways to react and respond to a cyber threat. Both attributes are essential from the security perspective.

### 4.3. Standards Collaboration and Co-Existence

This section discusses the recent collaboration between communication standards, 5G/TSN, 5G, and Wi-Fi 6 [4] supporting IIoT implementations. It is important to understand both security and communication standards and their co-relation as their synchronization plays an important role in designing a security strategy.

5G and TSN (IEC/IEEE 60802) is a joint project of IEC SC65C/WG18 and IEEE 802 for defining TSN profiles for industrial automation [4]. The IEC/IEEE 60802 profile specifies TSN applications for industrial automation and also provides 5G support guidelines. Ericsson had tested 5G’s integration with TSN providing E2E deterministic connectivity in a fully centralized model supported in 5G Phase 2, 3GPP Release 16, and time synchronization. In [67], the authors present an I4.0 use-case model implementing both 5G and TSN and identified limitations in the following areas: gap analysis, 5G/TSN configuration, E2E TSN stream configuration, and standardization issues. This indicates that regardless of implementing guidelines provided by standardization bodies, gaps within the environment still exist [68,69,70].

5G and Fiber: Fiber as a standalone option for connectivity may deliver the required performance metrics, i.e., speed, latency, and reliability, but 5G provides enhanced mobility and is enabled to resolve the last mile problems. The production environment must not consider 5G as a replacement to fiber, since it may require underlying fiber support in situations of small cell deployments and 5G radios [49].

5G and Wi-Fi 6 (Co-Existence): Both standards are built on the same foundation in terms of immersive experience, and IoT scalability (high throughput, low latency, high capacity). If they are deployed in a single connected factory, both standards can co-exist and complement each other supporting different use cases (indoor vs. outdoor), remote working, etc. Wi-Fi 6 [71] is considered as an optimal choice for indoor networks (with improvements in speed, latency, and higher density of connected devices, ideal for indoor enterprise networks and areas where access points can serve more users). 5G has not fully rolled out yet; however, the early use cases, which include fixed wireless (broadband backhaul), connected cars, drones, etc. make it a preferred method for outdoor networks. With the increased number of IoT devices in the coming years, alignment between 5G and Wi-Fi 6 will provide promising outcomes in terms of flexibility/mobility, increased productivity, and user experience.

5G and M2M: I4.0 recognizes the potential of 5G technology for M2M communications and is shaping the underlying systems that are 5G enabled [72]. The Open Mobile Alliance (OMA) protocol LwM2M is a step in this direction. LwM2M was designed for low data, low power, and constrained resource devices with cellular technologies such as LTE-M and NB IoT in mind. The EU’s project called Mobile and Wireless Communication Enablers for the 2020 Information Society (METIS) [38] developed a user-centric 5G system concept delivering the three generic services, eMBB, URLLC, and mMTC.

Another standard discussed here was ETSI OneM2M, which is a global standard that covers requirements, architectures, API specifications, security solutions and interoperability for M2M and IoT Technologies. OneM2M aligns with ATIS, ETSI, and collaborates with Qualcomm. At the service layer [21] it is interoperable with the NIST Cybersecurity Framework Protecting Information and System Integrity in ICS Environment Project, NIST 1108, NIST 7628, NIST SP 800-82 Rev 2, NIST SP80053 Rev 5 [34], IEC 62443/ISA 99, ISA 95, and IISF [63,64]. It was mentioned in [21] that IEC 62443 has blind spots, the authors suggest filling these gaps by implementing ETSI OneM2M (ETSI—TS 689 and TS 690) as both standards have the ability to co-exist and collaborate. Besides this, the NIST Cybersecurity Framework and ISO 27002 also complement each other in terms of information security.

## 5. Overview of Work on Standards, Cybersecurity, and Communications Related to I4.0

Since cybersecurity is a vast area, only works relevant to the author’s research in the context of IIoT/I4.0 were reviewed as presented in Table 3. This table outlines existing research in this space under three main headings, Standards, Cybersecurity and Communications. The authors reviewed over 90 papers published between the years 2016–2021, out of which 52 fit within the scope of this research, which is, securing M2M communications in the IIoT.

The papers [75,76] discuss how implementing security standards and controls increase trust, visibility, and integrity of the industrial environment. However, these standards need to be aligned with the manufacturing environments objectives and operational and functional requirements. As per [36,78,79] different wireless communication standards can co-exist and be deployed to attain agility and flexibility on the production floor. Authors in [50] point out limitations in existing standards, whereas [5,15,74] underline IT/OT convergence and interoperability issues [28,41]. The use-case examples presented in [6,11,77] bring attention to wireless communication security and performance metrics for I4.0 and [42,47] provide methods for improving M2M/IoT security at the component level. The authors of [25,44,81,82] indicate different cybersecurity issues (data integrity, dependability metrics) and risk parameters [72,73,74,75,76,77,78] which IIoT [70,82] might be susceptible to due to the 5G threat landscape [44,56,83,84] and SDNs [4,26]. Furthermore [5,51,96,97] discuss the architectural challenges associated with IoT/M2M communication using wireless standards [51,57,60].

The papers reviewed by the authors show existing cybersecurity threats and complexities with IIoT standardization. The volume of works also shows that research is being actively conducted in this domain as issues with security have not been fully resolved yet.

## 6. IT/OT Convergence and Issues

As the OT devices become universal intelligent devices, it exposes I4.0 to new security risks as it involves thousands of IoT devices communicating within the environment [3,46]. Each connection is a potential entry and how the industrial environment is protected may determine how strong/weak it is. Based on ENISA’s 5G threat classification, many of the threat vectors would be novel and go unidentified if not closely assessed and monitored. Both IT and OT implement different standards but with the new IIoT concept where any data should be available/accessible/retrieved/stored to and from anywhere, the best practices do not work sufficiently to protect the environment. IEC 62443 assists the IT/OT convergence but the standard itself is complex and leads to alignment issues. Some of the issues related to IT/OT convergence are discussed in [14,18,19,60,98]. They include outdated legacy systems with custom configurations and limited security, different security levels for IT/OT, increased security stakes, and IT/OT accountability and productivity risks. Limited or no interaction between the IT/OT teams and a lack of understanding of the security protocols of one another’s domain where different priorities and practices exist. Air-gapped or isolated OT systems with a lack of visibility and threat detection mechanisms are now connected to IT systems [18,20]. Successful infiltrations and OT breaches have been presented in Figure 3.

IT/OT systems involve different security services in the CIA triad, these services are categorized based on (a) priority differences, (b) standards and assessment methods, (c) tools and techniques, and (d) different technical teams. Due to lack of understanding and IT/OT convergence, [59] states that ICS security ranks among the bottom 2 in the security domain. ICS security is vital and if compromised it can lead to absolute downtime and freezing of operations. The same survey also mentioned that around 71% of the OT cybersecurity threats go unattended for up to 3 months. Some of the open research challenges in the IIoT IT/OT cybersecurity convergence are creating value in the connected factory, convergence to simplify process control, creating real-time visibility, and no code applications [59]. For securing the fully connected factory, it is essential to achieve uniformity and interoperability in all key areas such as assets, endpoint, and network protection, security monitoring, and reporting and secure remote access.

- IT/OT Priorities and Perspectives: Both IT and OT have different objectives [99] in terms of security (see Table 4). For IT, the metrics are assessed as CIA, whereas for OT the metrics are prioritized as AIC. “*IT and ICS networks are increasingly intermingled, the specialists on either side often do not fully understand each other’s operating concerns. This may result in a wide range of policy and controls gaps and change and asset management processes that have not matured to match the degree of integration that exists*” [17].

**Table 4:** IT/OT priorities.

IT	OT
Prioritize confidentiality over availability	Availability, efficient, and deterministic
Burden to manage more and more connected devices	Machine and processes—100% uptime
Factory and office ethernet (segmentation concerns)	Shorter response time/resilient

**Figure 6:**
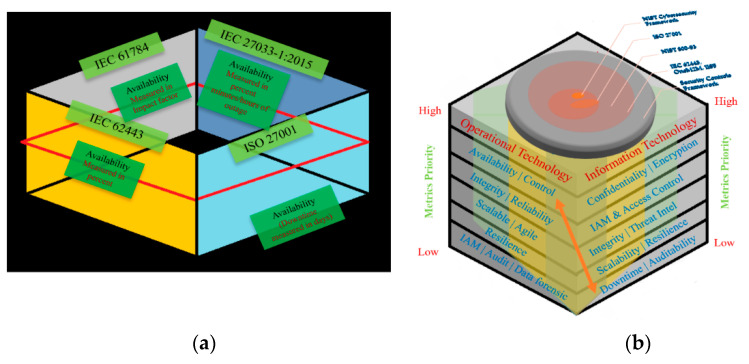
(**a**) The different standards within the manufacturing environment with the same metric. (**b**) Necessity to align cybersecurity standards and security controls.

For securing OT (ICS/SCADA) systems, availability secures the top place in the security objectives priority list, since it focuses on 100% uptime, visibility, and controlling the critical infrastructure. As per [43], the OT systems must allow systematic degradation (in emergency situations) in which the operations are transferred to manual status and control. Next comes integrity, which provides assurances to the operators that the data transitioning/received from various IoT-based devices is not manipulated or altered. ICS integrity is critical to OT security and the priority is to detect various security events and incidents, such as failure of devices and services exhausting resources early [100]. “*Confidentiality is not as important as the availability and integrity because the data received from sensors, PLCs and RTUs are used, whereas the data transitioning is state-based and only valid for that specific time, it is then discarded after processing and stored in historian servers. For instance, the living time for physical data to be processed is very short and the period is between two processed physical data as short as milliseconds in contract with the traditional IT data where credit card details are effective for many years* [101,102]”.

b. Standards and Assessment Methods: The feasibility of hybrid security standards has already been discussed and it is important to note that the majority of standards include and depend on similar metrics such as availability, reliability, integrity, and scalability, but have different methods and metrics (weightage/key performance indicator (KPI)) to measure them (see Figure 6a,b). For example, IEC 62443 is implemented for IT/OT security and has an availability metric, ISO 27001 (implemented for information security). IEC 61784 (industrial communication network profile) [103] and IEC 27033-1:2015 (IT security techniques—network security) [58] also consist of the availability metric.

Now if the availability metric is assessed differently (in terms of preference, weightage, and assessment criteria), it will not consider the security risks at the ICS level precisely leaving disparities between the IT and OT environment. Table 5 presents some of the security and communication standards implemented in the hybrid mechanism at the ICS level. It also depicts how each standard focuses on different aspects/areas for security controls. Since the availability metric holds a greater significance in IIoT, the authors recommend cross-referencing/linking it across different standards (using it) and assessing it with the same/consistent weightages/KPIs. For example, if availability is measured in percentage, it will be easier to assess and compare the risk threshold in cross-domain (IT and OT) systems. This method will also contribute toward aligning and improving ICS security.

In [104], the authors compared the security requirements of ISO 27001, NIST 800-53 [34], and IEC 61511 [105] to check whether ICS/SCADA safety is considered by the above standards. The comparison was made to verify whether the existing security objectives were sufficient or not. The security objectives in ISO 27001 and NIST 800-53 were inadequate and did not reflect the uniqueness of the nature of ICS/SCADA systems, but the safety requirements of IEC 61511 matched the common security controls. Henceforth, a need for new security objectives and safety is required for OT systems.

c. Separate tools and techniques deployed for measuring the IT and OT security controls as mentioned before.
d. Different technical teams and skill sets develop a wide security gap, as both IT/OT operators lack insights on the security aspects of each other’s domain [17]. This lack of qualified/trained skill set leads to compromised security as the team may fail to identify, understand, and assess the security issues associated with novel communication networks.

I4.0 lacks IT and OT convergence at the operational level, as mentioned before both IT and OT assess the security metrics differently leaving to vulnerabilities, which if identified and exploited by hackers may lead to major cyber threats such as DoS, loss of control, and defected products. Existing IT/OT security gaps are outcomes of standards alignment and interoperability issues. If each applied standard is assessed explicitly, it will fail to detect the security risks. As shown in Figure 6, each standard validates the cyber threat landscape from a different aspect (i.e., ICS security, communication protocols, information security, etc.) therefore, the security metrics must be mapped for securing M2M communications.

### Securing Industrial Control Systems

Security breaches within the IIoT environment ICS and SCADA (supervisory control and data acquisition) have been discussed previously and were subject to absolute failure/disruption at the OT level. In [14], the authors state that IIoT implementing SIEM (security information and event management) services, IDS (intrusion detection systems), IPS (intrusion prevention systems), and firewalls within ICS only assist in security at the network level. They do not counter attacks to the OT where critical processes rely on speed and availability because by implementing it at those levels the latency between critical processes can be increased. Even after breaches take place, identifying, assessing, and resolving it is another challenge. For securing the ICS [74], it is important to understand and implement solutions at various levels. These recommendations are generic and each industrial use-case may require different levels of implementation. Some of the challenges that ICS/SCADA systems are susceptible to are discussed below.

Securely extracting data from legacy (ICS/SCADA) systems is one of the biggest challenges addressed within the IIoT/I4.0. Some of the reasons for this are [105,106,107] old and basic architecture designs where security was not considered, with messages transmitted between remote connections internally without using encryption techniques, lack of patching system, securing ICS/SCADA remote connections which are scattered (sensors, actuators, PLCs, etc.), IT/OT incompatibility, and OT based-in remote locations with limited network connectivity. It also depends on what type of data needs to be extracted such as program running on PLC, Taglist, software, hardware, etc. Some commercial IoT-based software’s, like IIoT Edge Device, IndyaSYS technologies, Kosmos, allow data extraction from PLCs but the level of security of these tools has yet to be analyzed. 

**Figure 7:**
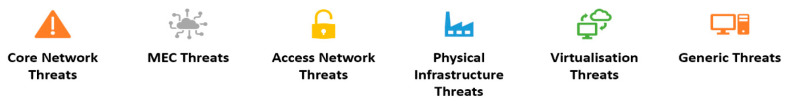
ENISA thematic landscape SDN/5G.

Achieving E2E security at the ICS level is important. This can be accomplished by implementing industrial blockchain technology to meet the required levels of security, openness, and decentralization [37]. This type of security has been implemented in TheConnectedFactory [108]. Besides Blockchain, there are also other methods to provide E2E security such as encryption techniques, authorization, access control, monitoring, etc. to mitigate the threats categorized by the ENISA thematic landscape 5G/software defined network (see Figure 7 below).

Table 6 illustrates the core attributes required for IT/OT security and mechanism of different standards supporting different security metrics. The complexity of M2M systems and security cannot merely be achieved by hybrid standards and security control implementations. The core security factor remains in designing and implementing a security strategy aligned with IIoT functionality and business needs. Section 7 introduces the author defined unified IIoT standards roadmap for aligning and converging the IT/OT domain.

To countermeasure the cybersecurity issues and challenges addressed in Figure 2, the authors have provided a roadmap for identifying the right standards, aligning, and implementing them to mitigate security gaps. The authors reviewed a wide range of research papers in cybersecurity, industrial wireless networks, security and standards space and it was identified that these issues still exist and have not been fully resolved to date.

## 7. The Unified IIoT Standards Roadmap

**Figure 8:**
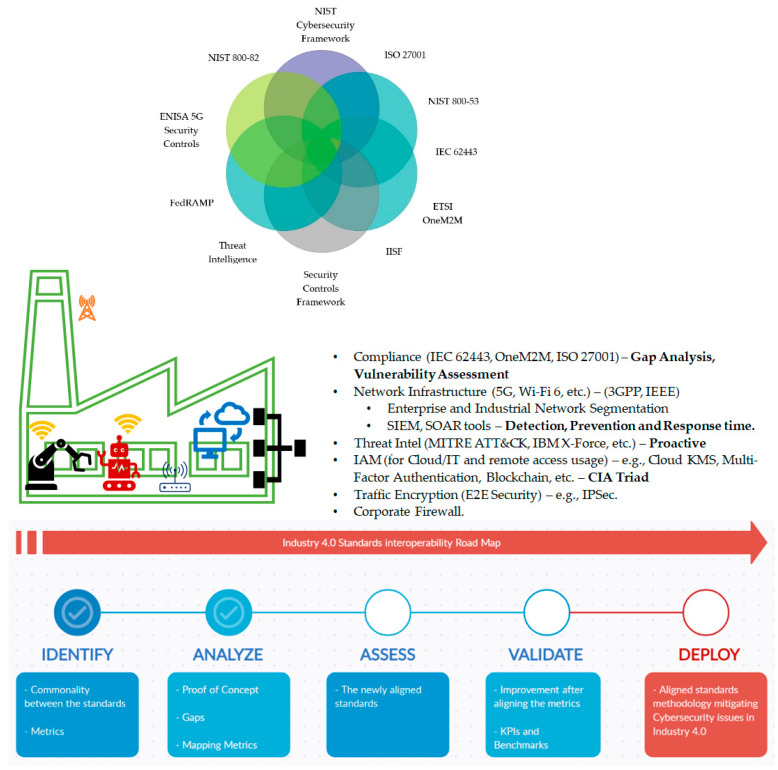
Unified IIoT standards roadmap.

A universal approach is required to secure and standardize the fully connected and intelligent manufacturing environment. Apart from IT/OT issues, IIoT vulnerabilities may also stem from areas such as (i) lack of required skill set, (ii) process and organizational issues, etc. The (author designed) unified approach assists in bridging the gaps between different communication standards, security objectives, and other areas which affect the overall cybersecurity domain. Figure 8 presents the I4.0 unified standards interoperability roadmap.

Compliance between different standards (hybrid standards) is necessary for achieving an independent/interoperable environment. Since common metrics used in hybrid standards are assessed in a similar manner, the gaps, and vulnerabilities shift from unknown (reactive) to known (proactive) risks. Section 4.3 highlighted gap analysis in 5G and TSN collaboration which will be mitigated by mapping the following security standards (IEC 62443, OneM2M, ISO 27001, etc.).

The IIoT/I4.0 network infrastructure involves the use of heterogeneous networks. Solely relying on a single communication network may limit the performance metrics and future growth of the industry. Since network segmentation [110,111] is considered as a good security practice, it can be achieved by implementing BP1-CPNI [109], IEC 62351 [112], and NIST 800-82. Whereas the security standards (NIST 800, ISO 27001, etc.) will provide guidance on deploying the standards. At this stage, the authors highly recommend I4.0 production environment designers to select and assess the standards based on their predefined objectives/targets, as this is the only that way the standards will provide promising outcomes and identify potential security risks/gaps.

The next step is to strategize and prioritize ICS/SCADA and IoT devices based on the level of protection required. The critical ones must be considered as top priorities and traffic should be filtered between different zones. Successful cybersecurity attacks at this layer will not only cause DoS and financial/asset losses but may also alter the production quality of the product or manufacture defective products causing safety issues. Implementing SIEM and SOAR tools at this layer will provide insights on the reactive approach to detect, prevent, and quickly recover from ICS attacks/failures [8]. At this point, Table 6 can act as a reference chart for analyzing the precision and effectiveness of standards and security controls. The table was adapted from [109] and can be extended or modified based on the production environments operations and functionality.

Threat intelligence provides a proactive and defensive edge in identifying the potential threats and attacks that the environment is vulnerable to. Implementing the MITRE ATT&CK framework [41] is fully justified at this level, as the comprehensive matrix includes different techniques and tactics used by cybersecurity assessment teams. The MITRE framework aligns with the IEC 62443 cybersecurity standard and assists the environment in defending itself. This framework classifies the cyberattacks with an impact factor/weightage assisting the environment in assessing the risk it may be exposed to. It is a proactive defensive approach allowing IIoT/I4.0 to identify gaps and prioritize the likely threats based on risk factor weightage (high/medium/low). It also provides additional benefits in terms of attack tolerance [8] and mapping risk and vulnerability assessment (RVA) to the ATT&CK framework.

In a fully connected universal machine environment, it is compulsory to deliver secure data transmission. For this aspect, identity and access management tools and traffic encryption (E2E security) assist in mitigating security issues related to IT/OT convergence, secure data extraction, and the security services (CIA).

Key management and cryptographic techniques are fundamental approaches for data security and protecting M2M networks from intruders. M2M networks are complex multi-hop communication systems where security needs to be provided by a smart system design [3]. For this reason, a suitable security system must provide E2E, hop-to-hop security, and key management services [113]. M2M networks involve low-end devices with limited resources and implementing costly security algorithms like asymmetrical cryptography does not fit the scope for IIoT [5].

There are different algorithms and techniques for key distribution and securing data aggregation mechanisms, but the authors focus on the role/contribution of CIA attributes while designing the security strategy which are discussed below.

Confidentiality and integrity focus on data/communication encryption techniques such as symmetric algorithms and hashing while key management systems assist in establishing strong keys and policy enforcement, managing threat impact, audit and freshness and data. IoT-based devices having authentication, access protection, and a security policy at this point will enable an ICS team to quickly identify and recover compromised devices on the industrial network when a breach takes place.

Kerberos [113,114] is also considered as a good option. Kerberos is a widely implemented network authentication protocol which uses symmetric key cryptography and third-party authentication for verifying user identities, making it hard for intruders to infiltrate. KDC is the fundamental part of Kerberos, consisting of three logical components, database of all principals and their associated encryption keys, authentication server, and the ticket granting server. Once the process is authenticated the entire communication is encrypted, ensuring privacy and data integrity [19]. Other viable solutions deployed at this stage are blockchain technologies, multi-factor authentication (MFA) systems like PassLogic, cloud-based KMS like AWS KMS, Azure Vault, and Cryptomathic KMS [115]. They all provide remote IAM services and E2E security.

The next phase involves deploying a firewall in the connected factory. Firewalls monitor and control network connections and communications based on predetermined security rules. IIoT firewalls partition traffic or data flows based on categorization of information risk (e.g., providing remote access to highly prioritized ICS and devices using firewalls and VPNs using IPsec (ESP tunnel mode) is considered sufficient as it counters unauthorized access). The IIoT production environment may implement more than a single firewall in order to protect both the IoT/M2M devices and production line (depending on the types of applications running and sensitivity of IT/OT data). Since one model does not fit all sizes, it is important that the types of firewalls (next generation based, hardware/software-based, cloud-based, etc.) implemented are aligned with the security objectives and IIoT technical requirements.

The roadmap also shows a hybrid security standards and control Venn diagram, designed by the authors, which aims at standards interoperability and convergence. This level of sophistication at mapping and alignment between security metrics can be achieved by implementing this roadmap. The phases mentioned at the bottom assist in identifying commonality between different standards (i.e., discussed in Section 6b), analyzing the type of production environment (i.e., physical factory, pod, etc.), operational and functional requirements, and accordingly map the security processes (see Table 6. ISO 27001 business continuity management, IEC 62443 IT/OT (ICS/SCADA) convergence, etc.). Assessing the customized/newly aligned standards meets the objectives. Verifying and validating the objectives are meeting the set goals (KPIs/benchmarks) and producing valuable feedback (which means that the standards and security controls are in conformance/mapped with the production environment). With these assurances the standards are deployed to mitigate the cybersecurity threat landscape to your systems.

The unified IIoT standards roadmap facilitates identifying and understanding the security objectives, i.e., how critical is the data? What channels must the data travel on? Which security attributes will it be exposed to? etc. By understanding the nature of ICS/SCADA operations and functionality, this approach will work as a roadmap in performing gap analysis from top to bottom, between the security standards, communication standards, and the threat intelligence frameworks. The authors state, if this roadmap is fully understood and implemented properly, it will enable IIoT/I4.0 in securing and mitigating IT/OT cybersecurity threat landscape and standardization issues.

## 8. Conclusions

This paper discusses various cyberthreats and risks which the IIoT/I4.0 is exposed to regardless of implementing cybersecurity standards and security protocols. IIoT/I4.0 is a fully connected autonomous factory which requires interoperable/universal standards bringing convergence and mitigating the security gaps that exist between the standards and controls. Each smart factory may have different service and security requirements and needs a customized security strategy solution aligning different cyber standards mitigating the threat landscape. The unified roadmap designed by the authors contributes toward (i) securing the heterogenous production environment, (ii) providing guidelines for identifying, assessing, and mitigating the novel cybersecurity-based threats, (iii) implementing different levels of protection, and (iv) providing IT/OT convergence and alignment. It also provides guidance for mapping and implementing diverse standards such as M2M communications, 5G, IoT, cloud, edge, etc. in harmony and eliminating the security risks and issues in IIoT/I4.0.

## Figures and Tables

**Table 1:** ENISA’s threat classification for IIoT.

Threat Classification	Security Service Breached/Compromised	Risk Impact
Nefarious activity/abuse (misuse, alter, steal, destroy information on targeted ICT systems, network, and infrastructure)	Confidentiality, Integrity, Availability	High
Eavesdropping/interception/hijacking (listening, interrupting, or gaining control of a third-party communication without approval)	Confidentiality	High
Disaster (accidental, force majeur, etc.)	Availability	Medium-High
Unintentional/accidental damages. This threat involves destroying, harming, or damaging people/property affecting the environment’s functionality or may lead to complete system failure (e.g., malware, misconfigurations, compromised hardware/software, etc.)	Integrity	High
Outage (service disruption)	Availability	High
Failure/malfunction (partially or complete lack of functionality of hardware/software assets)	Availability	High
Legal (legal issues related to third-party subcontractors, GDPR issues, lawful interception, e.g., unlawful surveillance, weaponization of interception, manipulation of information, etc.)	Confidentiality, Integrity, Availability	Medium-High
Physical attacks (exposing, leaking, falsifying, damaging, or gaining unauthorized access to ICT systems, networks, and infrastructure)	Confidentiality, Integrity, Availability	High

**Table 2:** Standards co-existence and comparison.

NIST 800-53 versus ISO 27001	NIST 800-53 [24] is security control driven (focuses on control mappings and baselines/best practices for information systems and organizations). ISO 27001 [33], which also refers to control categories with 114 Annex A Controls, is considered to be less technical and more risk focused for organizations of all types and sizes.
IEC 62443 versus ISO 27001	IEC 62443 versus ISO 27001: IEC 62443 [40,41] refers to information for implementing electronically secure industrial automation and control systems (IACS) at the IT/OT level, while ISO 27001 refers to information for implementing an information security management system (ISMS). IEC 62443 applies to products and ISO 27001 to organizations.
NIST 800-82 and IEC 62443	NIST 800-82 refers to securing ICS and SCADA systems. Both NIST 800-82 and IEC 62443 are applicable to the same type of industry and work on the same level (process control, collaborative robotics, additive manufacturing). However they differ in terms of security protection strategy, security management and security classification [42,43,44].
NIST 800-53 and Federal Risk and Authorization Management Program (FedRAMP)	Both of these standards complement each other in terms of governmental compliance fundamentals. NIST 800-53 [34] sets out prescriptive controls for data integrity, whereas FedRAMP [45] provides a standardized approach to security assessment, authorization and offers complimentary controls for cloud vendors.
Secure Controls Framework (SCF) [40]	An open-source, free to use framework which “*focuses on internal controls (cybersecurity, privacy-related policies, standards, procedures and other processes) designed to provide reasonable assurance that business objectives will be achieved and identifying and mitigating undesired events*” [40]. SCF has hybrid elements and covers different aspects of NIST 800-53, ISO 27002 and NIST Cybersecurity Framework (CSF). It also allows addressing multiple cybersecurity and privacy frameworks simultaneously. Based on the framework’s discussed attributes, it is understood to provide a comprehensive coverage.

**Table 3:** Synopsis of different cybersecurity standards and threats in IIoT.

**Standards—Objectives, Characteristics, and Limitations**	
[5]	addresses existing IACS cybersecurity issues related to IT/OT convergence. This paper also talks about the need for Standardizing IT/OT, IEC 62443, and limitations of existing security standards.
[73]	provides analysis of security standards for ICS.
[37]	compares existing industrial WSN standards in the following settings: (i) wireless standards for process automation (ZigBee, WirelessHART, ISA 100.11a, WIA-PA) and factory automation (WISA, WSAN-FA)—IEEE 802.15.4 (PHY and MAC Layer), (ii) network architectures for different wireless standards.
[15]	focuses on security breaches in ICS (SCADA systems) and forensic challenges in IIoT.
[74]	marks the limit of security and convergence in IT and OT systems.
[27]	notifies different community-based open standards such as MTConnect, OPC-UA and mentions limitations within the existing standards.
[36]	abstracts wireless standards IEEE 802.1 and use cases (i.e., industrial wireless and wireless access in medical environments).
[55]	quotes lack of compliance in IEC 62443 and the necessity for a standard compliant threat analysis process in IIoT.
[42]	discusses IEC 62443 implementation at the component level improving security for IoT devices.
[47]	elaborates IoT dimensions and sub-dimensions in IIoT architecture with reference to RAMI 4.0, IMSA, IVRA, IIRA, SME, F-CPS, and IoT ARM.
[4]	sets forth IEEE 802.1 TSN performance and security requirements supporting IIoT. (i) Time/mission critical data traffic supporting IIoT performance requirements, (ii) IEC/IEEE 60802 TSN for industrial automation, and (iii) 5G support for TSN and integration.
[61]	drafts IEEE standards in digitization of EU industry/advanced manufacturing and shows how IEEE TSN is enabled to deliver deterministic connectivity to time and mission critical IIoT-based applications over ethernet networks (IEEE 802.3).
[28]	discusses M2M standards (OneM2M, HyperCat, OMA Lightweight, ECLIPSE M2M, Weightless), cross-level standards (IEC 62443/ISA 99, ISO 9000, etc.) enabling technologies and key capabilities.
[75]	summarizes ETSI OneM2M architectures and standard M2M architecture for universal machines.
[76]	outlines smart manufacturing standards (ISO/IEC/JTC1) for CPS IT.
[77]	reviews intelligent M2M systems and complex event processing for M2M communications.
[78]	compares wired and wireless standards in terms of performance metrics (i.e., network security, reliability, visibility, cost, etc.).
[41]	encapsulates industrial cybersecurity standards (IEC 62443 for SCADA and industrial control system security). Focuses on ICS security attributes and challenges, NIST SP 800-82 ICS security, ISA 99—ISA/IEC 62443, industrial communication networks and system security.
[79]	compares industrial WSN standards (i.e., ZigBee, WirelessHART, ISA 100.11a) performance and security (authentication, integrity, encryption techniques) characteristics in industrial environment.
**Cybersecurity—Objectives, Characteristics, And Limitations**	
[25]	addresses the need of security solutions for data management systems in CPS.
[26]	examines 5G cybersecurity risk assessment (i.e., threat environment, vulnerabilities to the IIoT and its impact on assets, possible risk scenarios, security baseline).
[2]	summarizes the ENISA threat landscape for 5G networks.
[80]	highlights cybersecurity and privacy challenges in H2020-projects (i.e., ANASTACIA, SAINT, FORTIKA, CYBECO, CS-AWARE, ARIES, LEPS, etc.).
[81]	outlines a security policy-based approach implemented and tested in H2020 EU project ANASTACIA, showing its feasibility to mitigate cyberattacks.
[82]	gives the main points of securing 5G networks (EU toolbox), cybersecurity requirements, and risk assessment methodologies for the EU IIoT environment.
[83]	discusses security of existing industrial and manufacturing systems and compares manufacturing and IT systems in terms of (systems, operations, security, and impact metrics) IIoT dependability metrics versus security metrics. Security solutions: Standards (IEC 62443—IACS, ISO/IEC 27033:1:2015—IT network security, IEC 61508—electronics in industry, IEC 61784 -industrial communication networks, ISO/IEC 27000—information security management). It also suggests 20 different intrusion detection systems for process systems.
[44]	overviews IoT cybersecurity (objectives, risks, and threats) and standards landscape (cryptographic techniques, IAM, network security, etc.).
[84]	lays a foundation for security services, methodologies, and procedures to secure data transmission and M2M communications.
[56]	reviews securing communication channels architecture for software defined mobile networks (SDMN) and implementing IPsec tunnels for securing SDMN communication channels. The real test-bed results showed SDMN architecture secured the environment against IP-based attacks (i.e., DoS, reset, spoofing, replay, etc.) but it affected the performance (throughput, latency) levels.
[70]	provides analyzing of 5G threats (DoS, hijacking, security keys exposure, signaling storms, etc.) and possible solutions.
[85]	suggests new approaches for cyber–physical security (security Services (CIA), IT/OT convergence, security issues related to distributed manufacturing environments, manufacturing security enforcement device (MSED)—cryptographically ensuring data integrity).
[86]	demonstrated cybersecurity analysis based on reference architecture model (RAMI 4.0) and the VDI/VDE guideline 2182 (IT security for industrial automation).
[87]	outlines IoT/M2M Communications cybersecurity issues.
[88]	discusses security requirements and constraints in M2M communications and solutions to mitigate these risks.
[89]	provides a framework for the future development of IMT 2020 and beyond.
**M2M Communications In Industrial Wireless Networks—Objectives, Characteristics, And Limitations**	
[68]	summarizes security functions provided by 3GPP and 5G security architecture.
[57]	examines 6G networks, potential use cases, the 3D network architecture, and its key capabilities.
[8]	highlights ICS concerns (i.e., gaps between legacy equipment and new communications systems (retrofitting, interoperability, and reliability issues) between industrial requirements and wireless standards).
[72]	focuses on IIoT use cases, performance metrics, standards, and security. It also puts a light on 5G key technologies and challenges.
[90]	provides a 5G digital factory workflow, mapping the smart factory performance metrics with 5G capabilities.
[69]	discusses 5G-ACIA initiative and manufacturing solution architecture. The white paper also mentions more than 90% of OT networks are connected by wired technologies (PROFIBUS, PROFINET, EtherCAT, Modbus, etc.).
[91]	delivers IIoT proof of concept using IWC 3.7-3.8 GHz band having capabilities of 5G standalone network.
[60]	reviews 5G industrial connectivity trends driving the IT/OT convergence.
[92]	elaborates Wi-Fi 6 performance and technological differences between Wi-Fi 6 and 5G.
[93]	gives a summary of IEC and IWN standards.
[94]	runs through industrial wireless standards and limitations of WLANs/WWAN networks.
[95]	outlines IWN (standards, QoS, real-time algorithms), security and data fusion issues in I4.0.
[51]	discusses M2M Communications in 5G: state-of-the-art architecture, recent advances. It also mentions the following research challenges: M2M architecture (device, communication and server/application domain), standards (ETSI, 3GPP, IEEE), secured M2M communication collaborations (LOLA, 5GPPP SESAME, etc.) and 5G services for future M2M communication.
[53]	articulates the scope of 5G standardization and advanced wireless technology.
[96]	gives a synopsis of 5GPP and 5G empowering vertical industries/IIoT. It also describes the 5G architecture for distributed and flexible network functions.
[3]	provides knowledgeable insights on M2M System architecture and security (vulnerabilities, services (CIA), protocols and algorithms) issues.
[97]	mentions challenges associated with cellular M2M and capillary M2M communications using wireless standards. It also examines M2M and wireless standards—IoT, WSNs, M2M, and CPS.

**Table 5:** IIoT communication and security standards.

IEC 62443 Security Capabilities for ICS Components [46]	IEC 61784-1:2019 Industrial Communication Networks (Communication Profile) [103]	ISO 27001 Information Security Management Systems—Requirements (Annex A. Controls (C)) [33,101]	ISO 27033:1: 2015 Information Technology Security Techniques—Network Security [58]
ISA-62443-4-2, Security for IACS: Technical Security Requirements for IACS Components ISA/IEC 62443-4-1, Product Security Development Life-Cycle Requirements	IEC 61784-1:2019 (E) defines a set of protocol specific communication profiles based primarily on the IEC 61158 series, to be used in the design of devices involved in communications in factory manufacturing and process control [98]	Information Security Policies—2 C Organization of Information Security—7 C Human Resource Security—6 C Asset Management—10 C Access Control—14 C Cryptography/Encryption—2 C Physical and Environment Security—15 C Operations Security—14 C	Overview and concepts Guidelines for the design and implementation of network security Reference networking scenarios—threats, design techniques, and control issues
ISA/IEC 62443-3-3, System Security Requirements and Security Levels ISA/IEC 62443-3-2, Security Risk Assessment, System Partitioning and Security Levels	IEC 61784-2:2019, Industrial communication networks (Profiles Part 2): Additional fieldbus profiles for real-time networks are based on ISO/IEC/IEEE 8802-3	Communications Security—7 C System Acquisition and Maintenance—13 C Supplier Relationships—5 C Security Incident Management—7 C Business Continuity Management—5 C Compliance—8 C	Securing communications between networks using security gateways Securing communications across networks using Virtual Private Networks (VPNs)
ISA/IEC TR62443-2-3, Patch Management in the IACS Environment The key concept is the application of IACS security zones and conduits, which are introduced in ISA/IEC 62443-1-1, Concepts and Models	IEC 61784-5-2:2018, Industrial communication networks (Profiles Part 5-2): Installation of fieldbuses—Installation profiles for Communication Profile Families (CPF 2)		Securing wireless IP network access

**Table 6:** Various standards illustrating different aspects of ICS/SCADA security [109].

	BPI-CPNI [109]	NIST 800-82	NIST 800-100	NIST 800-48 & 800-97 [109]	ISO 27001	IEC 62443	IEC 61784	OneM2M	MITRE ATT&CK
Access control	●	●			●			●	●
Asset categorization and control			●		●				●
Business continuity management					●				
IT/OT (ICS/SCADA) convergence		●				●		●	
ICS/SCADA characteristics, threats, and vulnerabilities		●				●			●
ICS/SCADA security controls (management, operational, technical)		●				●	●	●	
Multi-connections to ICS/SCADA network	●							●	
Network architecture security	●					●		●	
Patch management strategies	●	●						●	
Physical and environmental security					●				
Physical and logical demilitarized zone (DMZ)	●	●							
Remote Access/IAM policy	●							●	
Security strategy			●		●				
Standards interfaces between different networks							●	●	
Logical segmentation on virtual LANs		●				●			
Physical segmentations	●					●			
Wireless network security				●		●	●		●
E2E encryption						●			

## Data Availability

Not applicable.

## Abbreviations

AES	Advanced Data Encryption Standard
AI	Artificial Intelligence
AIC	Availability Integrity Confidentiality
ARIB	Association of Radio Industries and Business
ATIS	Automatic Terminal Information System
CCSA	China Communications Standards Association
CEN CENELAC	European Committee for Standardization
CIA	Confidentiality Integrity Availability
CPS	Cyber–Physical Systems
CSF	Cybersecurity Framework
DLMS	Device Language Message Specification
DoS	Denial of Service
EAP	Extensible Authentical Protocol
eMBB	Enhanced Mobile Broadband
ENISA	European Network and Information Security Agency
ETSI	European Telecommunications Standards Institute
EU MS	European Member States
E2E	End-to-end
FedRAMP	Federal Risk and Authorization Management Program
GDPR	General Data Protection Regulation
IACS	Industrial Automation and Control Systems
IAM	Identity and Access Management
ICT	Information and Communication Technology
ICS	Industrial Control Systems
IDS	Intrusion Detection Systems
IEC	International Electrotechnical Commission
IEEE	International Institute of Electrical and Electronics Engineers
IETF	Internet Engineering Task Force
IIRA	Industrial Internet of Things Reference Architecture
IISF	Industrial Internet Security Framework
IoT	Internet of Things
IIoT	Industrial Internet of Things
IPS	Intrusion Prevention Systems
ISA	International Society for Automation
ISMS	Information Security Management Systems
ISO	International Organization for Standardization
IT	Information Technology
IWN	Industrial Wireless Network
JTC	Joint Technical Committee
KDS	Key Distribution Center
KMS	Key Management System
KPI	Key Performance Indicator
LTE	Long-Term Evolution
LWM2M	Lightweight Machine-to-Machine
MEC	Multi-Access Edge Computing
METIS	Mobile and Wireless Communication Enables for the 2020 Information Society
MFA	Multi-Factor Authentication
mMC	Massive Machine Type Communications
M2M	Machine-to-Machine
NB IoT	Narrowband Internet of Things
NIST	National Institute of Standards and Technology
NFV	Network Function Virtualization
OMA	Open Mobile Alliance
OT	Operational Technology
PDCA	Plan Do Check Act
PLC	Programmable Logic Controller
PLT	Power Line Telecommunications/Transmissions
QoS	Quality of Service
RAMI	Reference Architecture Model Industry 4.0
RTU	Remote Terminal Unit
RVA	Risk and Vulnerability Assessment
SCADA	Supervisory Control and Data Acquisition
SCF	Secure Controls Framework
SDN	Software Defined Network
SIEM	Security Information and Event Management
SLA	Service Level Agreement
SOAR	Security Orchestration Automation and Response
TIA	Telecommunications Industry Association
TISPAN	Telecoms & Internet converged Services & Protocols for Advanced Networks
TSN	Time Sensitive Networking
TTA	Telecommunications Technology Association
TTC	Telecommunication Technology Committee
URLLC	Ultra-Reliable Low Latency Communications
VPN	Virtual Private Network
WG	Working Group
3GPP	3rd Generation Partnership Project
5GPPP	5G Infrastructure Public Private Partnership
